# A Rapid UHPLC Method for the Simultaneous Determination of Drugs from Different Therapeutic Groups in Surface Water and Wastewater

**DOI:** 10.1007/s00128-012-0634-7

**Published:** 2012-04-10

**Authors:** Irena Baranowska, Bartosz Kowalski

**Affiliations:** Department of Analytical Chemistry, Chemical Faculty, Silesian University of Technology, 7 Strzody Str., 44-100 Gliwice, Poland

**Keywords:** UHPLC, Pharmaceuticals, SPE, Water samples

## Abstract

A SPE-UHPLC-UV method for the determination of 13 pharmaceuticals from different therapeutic groups in surface water and wastewater samples was proposed. The following three solid-phase-extraction (SPE) columns with polymeric sorbents were used as a pre-concentration step: the Oasis HLB (average recoveries 93.8 %), the Nexus (84.0 %) and the Bond Elut ENV (88.3 %). A reverse-phase UHPLC with a C_18e_ monolithic column and gradient elution program was used to obtain the best separations for all 13 drugs in short analysis time (3.4 min). The LOD range for determined drugs was 0.02–0.18 μg L^−1^, and the concentration range for drugs found in water samples was 0.06–0.90 μg L^−1^. The proposed method was used to analyze different water samples, mostly from rivers, and can be used as a monitoring tool for environmental pollution.

Pharmaceuticals are widely used in human and veterinary medicine and are present in various water samples because up to 95 % of the dose can be excreted or discharged directly into domestic wastewater (Farre et al. 2007). Moreover, most of the pharmaceuticals are not eliminated by wastewater treatment plants (WWTPs) due to insufficient technology for the removal of such contaminants. The non-eliminated pharmaceuticals from WWTPs reach ground waters and could be harmful to aquatic organisms even when they are present at low concentrations (ng L^−1^) (Hong et al. 2007). Although the documentation is limited, it is undeniable that this kind of water pollution could be harmful even for humans (Zhou et al. 2009).

Many pharmaceuticals have been found in wastewater treatment plant (WWTP) influents and effluents, rivers, and even in drinking water. Most of them were usually at low ng/L concentrations (Kasprzyk-Hordern et al. 2009; Mompelat et al. 2009). The most commonly used methods for the determination of drugs in water samples are GC or HPLC with MS or tandem MS–MS detectors (Gomez et al. 2007; Rodil et al. 2009). HPLC with DAD detection is rarely used (Gil García et al. 2009). However, most of these publications focused on drugs belonging to one therapeutic group. As a pre-concentration step, SPE procedures have been proposed (Grujic et al. 2009; Moldovan et al. 2009); however, some other techniques based on solid-phase and liquid-phase extractions have also been described (Es’haghi 2009). The development of techniques that provide faster analysis is one of the latest trends in analytical chemistry. The use of UHPLC for the determination of contaminants in environmental samples has become the most suitable analytical tool to improve analysis time, sensitivity and can significantly reduce labor costs (Gracia-Lor et al. 2010; Richardson 2009).

The aim of this work is to develop a fast, selective and sensitive method using UHPLC with UV detection and an SPE procedure to provide sample enrichment for the determination of 13 acidic, neutral and basic drugs belonging to different therapeutic groups. The drugs examined included paracetamol (PAR), sotalol (SOT), metamizole (MTZ), salicylic acid (SAL), metoprolol (MET), aspirin (ASP), propranolol (PRO), prednisolone (PRE), carbamazepine (CBM), carvedilol (CAR), dexamethasone (DEX), naproxen (NAP) and diclofenac (DIC). The drugs selected are widely used and were found in many water samples in relatively high amounts (μg L^−1^ for β–blockers, NSAIDs and the anticonvulsant carbamazepine). The presence of prednisolone and dexamethasone in water samples has not been confirmed in the literature; however that class of drugs has been distinguished as a potential environmental risk (Piram et al. 2008). The procedure for the pre-concentration and simultaneous determination of the 13 drugs selected has not been described in the literature. The use of UHPLC results in shorter analysis times and reduced labor costs, which is important for routine analysis. The method developed herein has been applied to water samples, mostly from Poland.

## Materials and Methods

Metamizole monohydrate was purchased from Riedel-de Haën (Seelze, Germany), and paracetamol was purchased from Fluka BioChimika (Darmstadt, Germany). Diclofenac sodium, aspirin, salicylic acid, carbamazepine, naproxen, sotalol hydrochloride, metoprolol tartrate, propranolol hydrochloride, carvedilol, prednisolone and dexamethasone were all purchased from Sigma-Aldrich (Milwaukee, WI). HPLC grade acetonitrile, water, methanol and formic acid were acquired from Merck (Darmstadt, Germany), and analytical grade methanol was bought from POCH (Gliwice, Poland).

Stock solutions of carvedilol and aspirin were prepared by dissolving standard (10 mg) in analytical grade methanol (10 mL). Stock solutions of the rest of the pharmaceuticals were prepared by dissolving the standard (10 mg) in a mixture of distilled water/methanol (10 mL, 50/50, v/v). All of the stock solutions were stable for at least 3 months at −18°C, except for aspirin. The stock solution of aspirin was stable for 2 weeks. Working solutions were prepared daily by mixing the appropriate volume of each stock solution with a mixture of distilled water/methanol (90/10, v/v).

The UHPLC system included an L-2350 column oven (LaChrom Elite, Merck Hitachi), a L–2200U autosampler, two L-2160U pumps and a L-2400U UV detector (LaChrom Ultra, Merck Hitachi). A Chromolith^®^ Fast Gradient monolithic C_18_e reverse-phase column (50 mm × 2 mm) from Merck was used. The data were collected with EZChrom Elite software. The solid-phase-extraction (SPE) was performed using J.T. Baker spe-12G (Deventer, Netherlands).

Three different SPE columns were used for the sample extraction procedure for the water samples. These columns included a NEXUS column (6 mL, 200 mg, Varian), a Bond Elut ENV column (6 mL, 500 mg, Varian) and an Oasis HLB column (6 mL, 500 mg, Waters). All of the columns were conditioned (except for the non-conditioned NEXUS column) with methanol (6 mL) and distilled water (6 mL) at pH 7 and a flow rate of 1 mL min^−1^. One liter of distilled water spiked with all 13 pharmaceuticals (2 μg) was passed through the columns at a flow rate of approximately 5 mL min^−1^. After the sample had passed through, each column was dried for 10 min. Then the analytes were eluted with methanol (5 mL), evaporated to dryness under a stream of nitrogen, and reconstituted in a mixture of distilled water/methanol (1 mL, 90/10, v/v). Then, 2 μL of the obtained extracts was injected through the auto sampler into the UHPLC system. This procedure was also tested on 1 L of tap water spiked with all 13 drugs (2 μg).

Water samples were collected mostly from mainstream rivers in Poland and one from the Czech Republic. All samples were stored at 4°C until analyzed (48 h).

Seven surface water samples were collected from the Vistula River from the following different cities: Skoczow, Cracow, Kazimierz, Warsaw, Bydgoszcz and two from Torun [before the Old City (Torun 1) and after the Old City (Torun 2)]. The rest of the water samples were collected from the following rivers: the Vltava (Prague), the Oder (Wroclaw), the Brda (Bydgoszcz), the Warta (Zawiercie and Czestochowa), the Krzywa (Bielsko-Biala), the Klodnica (Gliwice), the Potok Toszecki (Toszek), the Mala Panew (Zawadzkie) and the Troja (Nowa Cerekwia). One sample was collected from the wastewater treatment plan (WWTP) effluent from Bielsko-Biala.

The best separations for all pharmaceuticals were achieved on the C_18e_ monolithic column at a temperature of 20°C using UHPLC equipment and a UV detector. A gradient comprised of two solvents, where solvent A was 0.1 % formic acid and solvent B was acetonitrile was optimized to obtain the best separations in the shortest possible time. The column eluent was analyzed at the characteristic detection wavelength for each pharmaceutical using a UV detector.

## Results and Discussion

**Fig. 1:**
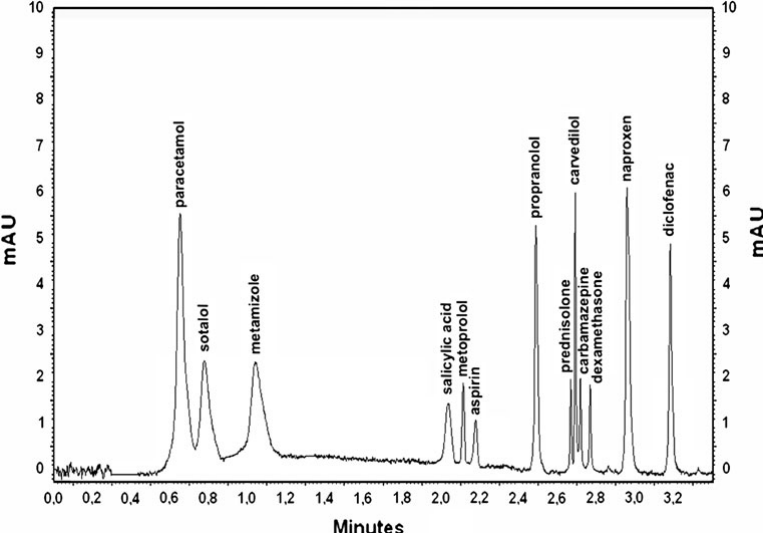
The chromatogram of the standard mixture containing 2 μg mL^−1^ for all drugs performed on the UV detector

For the determination of the investigated drugs in water samples, a gradient elution was used. The best separations were achieved on the C_18e_ monolithic column (Chromolith^®^ Fast Gradient) with a two-solvent gradient elution consisting of 0.1 % formic acid in water (A) and acetonitrile (B) (Table 1). All 13 drugs were eluted in less than 3.2 min with satisfactory separations (Fig. 1). The retention times, standard deviations and analytical wavelengths are shown in Table 2. The short analysis time allowed examination of a large number of samples within a single working day, which can reduce labor costs.

**Table 1:** The best gradient elution program: A: 0.1 % formic acid in water, B: acetonitrile

Time (min)	Solvent		Flow rate (mL/min)
	A (%)	B (%)	
0.0	100	0	2.0
1.0	98	2	2.0
2.0	80	20	2.0
3.0	40	60	2.0
3.5	20	80	2.0

**Table 2:** Wavelengths, retention times, standard deviation and coefficient of variation (n = 6)

Drug	Wavelength (nm)	Retention time (min)	Standard deviation (min)	Coefficient of variation (%)
Aspirin	229	2.175	0.003	0.14
Carbamazepine	215	2.713	0.008	0.37
Carvedilol	227	2.685	0.011	0.52
Dexamethasone	241	2.783	0.007	0.34
Diclofenac	275	3.161	0.006	0.26
Metamizole	259	1.020	0.011	0.49
Metoprolol	227	2.108	0.008	0.36
Naproxen	231	2.973	0.007	0.31
Paracetamol	241	0.648	0.009	0.43
Prednisolone	241	2.658	0.008	0.37
Propranolol	227	2.495	0.009	0.39
Salicylic acid	241	2.048	0.007	0.34
Sotalol	227	0.816	0.013	0.59

Three SPE columns with polymeric sorbents were tested for the recovery efficiency of the pharmaceuticals investigated. The selected columns were the Bond Elut ENV, the NEXUS and the Oasis HLB. The Oasis HLB column and the ENV column have been widely used for the enrichment of analytes from water samples. The non-conditioned Nexus column is a new sorbent that has not been described in the literature for the enrichment of analytes from water samples.

Samples of distilled water with each pharmaceutical (2 μg L^−1^) were passed through each column, and the recovery efficiency was determined. Then, the procedure was tested on 1 L tap water samples. The results for the recovery efficiency are presented in Table 3. The recoveries obtained were on the same level in the distilled water and tap water for each pharmaceutical, respectively. Most of the drugs on each column tested were recovered with over 90 % efficiency. Only carvedilol and paracetamol using the NEXUS column, paracetamol using the ENV column and metamizole using the HLB column were recovered with efficiency below 70 %. Despite the lower efficiency, the precision for the drugs tested was satisfactory for the proposed procedure. The highest recovery efficiencies were achieved using the Oasis HLB column (over 90 % for 11 drugs, mean 93.8 %). Nevertheless, the Bond Elut ENV (mean 88.3 %) and the NEXUS (mean 84.0 %) column also gave satisfactory recovery efficiencies and can also be used for the enrichment of analytes from water samples in other mixtures.

**Table 3:** Recoveries (n = 6) for all pharmaceuticals in 1 L of spiked distilled and tap water

	Recoveries (SD) (%)					
	Distilled water			Tap water		
	ENV	NEXUS	HLB	ENV	NEXUS	HLB
Aspirin	90.1 (5.8)	75.3 (4.2)	93.4 (4.8)	83.2 (2.6)	70.7 (3.6)	91.8 (2.9)
Carbamazepine	98.4 (2.2)	99.1 (4.7)	101.9 (3.5)	102.0 (3.3)	102.3 (5.1)	97.0 (0.6)
Carvedilol	87.7 (1.5)	37.4 (4.9)	98.2 (2.3)	95.4 (3.4)	39.6 (1.4)	103.4 (5.8)
Dexamethasone	103.6 (1.7)	101.7 (6.8)	95.8 (1.3)	98.9 (4.9)	104.2 (6.3)	99.0 (2.8)
Diclofenac	99.7 (1.4)	97.7 (1.5)	102.2 (3.1)	96.3 (1.6)	98.2 (2.2)	102.4 (1.7)
Metamizole	82.2 (8.1)	89.7 (5.0)	53.5 (1.9)	92.1 (1.4)	88.2 (5.4)	53.9 (8.5)
Metoprolol	90.2 (7.6)	87.5 (7.1)	101.6 (3.4)	105.7 (6.1)	99.6 (2.8)	99.1 (8.6)
Naproxen	101.1 (2.5)	106.9 (9.0)	102.6 (3.4)	100.3 (3.0)	102.7 (4.7)	99.4 (3.1)
Paracetamol	36.5 (1.5)	38.7 (2.1)	72.0 (7.2)	38.7 (5.0)	39.6 (2.2)	70.5 (4.2)
Prednisolone	98.9 (4.0)	96.5 (3.0)	102.7 (5.5)	102.3 (8.7)	98.4 (2.9)	100.8 (1.8)
Propranolol	79.6 (1.8)	99.7 (4.5)	97.3 (5.4)	100.1 (4.5)	98.5 (2.8)	94.7 (4.1)
Salicylic acid	78.1 (4.9)	72.6 (1.7)	100.5 (6.7)	95.1 (9.4)	67.4 (4.9)	95.7 (5.9)
Sotalol	102.2 (3.2)	89.0 (7.3)	97.9 (5.2)	98.3 (1.7)	86.0 (6.9)	92.0 (9.3)

The recovery efficiencies for the drugs selected, described separately or with other drugs, are generally in agreement with literature data found for the Oasis HLB column, except for prednisolone, dexamethasone and carvedilol, which recovery efficiencies on the Oasis HLB column in water samples were not found in literature data. The recovery efficiency data corresponding to the Nexus and the Bond Elut ENV for these drugs in water samples are not known. However, the recovery efficiency data using the polymeric sorbent ENV from different manufacturers are known only for some of the drugs used in this study.

Standard curves for the pharmaceuticals used in this study were determined using the following linear regression: *y* = *ax* + *b*, where *y* is the peak area, *a* is the slope, *x* is the respective concentration and *b* is the intercept. The parameters of the calibration curves for all pharmaceuticals are presented in Table 4. The limit of detection (LOD) and limit of quantification (LOQ) were determined using the parameters of the standard curves and then recalculated using the appropriate recovery level of each drug in 1 L of tap water. The LOD values were determined as LOD = 3.3 *s*/*a*, where *s* is the standard deviation of intercept (*S*_*b*_), and *a* is the slope. The LOQ values were calculated as LOQ = 3LOD. For all drugs, values of LOD and LOQ under 0.5 μg L^−1^ were achieved. For some drugs, values of LOD and LOQ were under 0.1 μg L^−1^. The low values of LOD and LOQ allowed us to detect and quantify some of those drugs in surface water samples.

**Table 4:** Parameters of calibration curves, linearity ranges and LOD and LOQ values

Drug	Linear range (μg mL^−1^)	Slope (a)	S_a_	Intercept (b)	S_b_	S_xy_	R^2^ (n = 6)	LOD (μg L^−1^)	LOQ (μg L^−1^)
Aspirin	0.29–10	10627	67	−1480	308	552	0.9998	0.10	0.29
Carbamazepine	0.53–10	9579	89	−3501	498	676	0.9998	0.18	0.53
Carvedilol	0.41–10	13178	178	−541	819	1468	0.9994	0.14	0.41
Dexamethasone	0.06–10	11351	18	−183	71	163	0.9999	0.02	0.06
Diclofenac	0.06–10	8690	10	176	41	94	0.9999	0.02	0.06
Metamizole	0.45–10	3336	31	1492	143	260	0.9997	0.15	0.45
Metoprolol	0.24–10	9476	48	−1041	226	411	0.9998	0.08	0.24
Naproxen	0.06–10	66890	99	−961	397	935	0.9999	0.02	0.06
Paracetamol	0.08–10	34863	62	344	254	545	0.9999	0.03	0.08
Prednisolone	0.06–10	9227	15	−25	59	136	0.9999	0.02	0.06
Propranolol	0.06–10	195305	326	−5520	1262	2905	0.9999	0.02	0.06
Salicylic acid	0.19–10	14915	63	−696	295	538	0.9999	0.06	0.19
Sotalol	0.39–10	10126	78	−180	365	665	0.9997	0.13	0.39

**Fig. 2:**
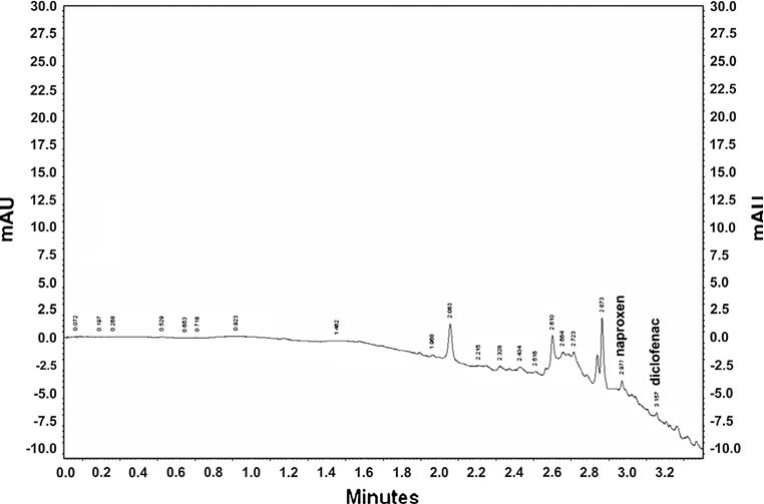
The chromatogram of the Vistula river extract from Bydgoszcz after SPE procedure (NAP: 0.19 μg L^−1^, DIC: 0.14 μg L^−1^)

The SPE-UHPLC-UV method was applied to the simultaneous determination of all 13 pharmaceuticals in water samples (Table 5). The blank samples from tap water and the appropriate recovery efficiencies were taken into account when calculating concentrations. The identification of drugs in real water samples was performed by comparing the retention times of standard solutions using the standard addition method. The only pharmaceuticals found in the majority of water samples were diclofenac and naproxen mostly at concentrations under 0.30 μg L^−1^. Diclofenac was found in higher concentrations only in Oder (Wroclaw) and Warta (Czestochowa). Naproxen was found in higher concentration in Klodnica (Gliwice). Salicylic acid was found in eight water samples and quantified in six with concentrations of 0.19–0.50 μg L^−1^. Aspirin, paracetamol and propranolol were detected in only 3–4 water samples. Metoprolol was found only in WWTP effluent with a concentration of 0.27 μg L^−1^, and metamizole was found only in Oder (Wroclaw) with a concentration of 0.90 μg L^−1^. Carvedilol, carbamazepine, dexamethasone, prednisolone and sotalol were not found in any of the analyzed water samples. In most of the water samples, two or three pharmaceuticals were found from those determined in this paper; however, five different pharmaceuticals were found in WWTP effluent from Oder (Wroclaw) and Vltava (Prague). Those water samples were collected near the WWTPs, which could probably explain the presence and relatively high concentrations of these drugs (in Oder over 0.40 μg L^−1^ for aspirin, diclofenac and metamizole). In some smaller rivers (Potok Toszecki and Mala Panew) only one drug (diclofenac and naproxen, respectively) was present, which could be explained by the fact that in the nearest region has no WWTPs. All pharmaceuticals determined were found in real water samples at low sub-micro levels (0.06–0.90 μg L^−1^); however, in only a few cases the concentrations were higher than 0.30 μg L^−1^. The chromatogram of sample extract from the Vistula river (Bydgoszcz) is presented in Fig. 2.

**Table 5:** Concentrations and standard deviations (μg L^−1^) of pharmaceuticals in different surface water samples (n = 3)

	Concentration (SD) (μg L^−1^)												
	ASP	CAR	CBM	DEX	DIC	MET	MTZ	NAP	PAR	PRE	PRO	SAL	SOT
WWTP B.-B.	–	–	–	–	0.16 (0.03)	0.27 (0.07)	–	0.11 (0.02)	–	–	<LOQ	0.27 (0.08)	–
Vistula Skoczow	–	–	–	–	0.09 (0.02)	–	–	–	<LOQ	–	–	–	–
Vistula Krakow	–	–	–	–	–	–	–	0.30 (0.04)	–	–	–	–	–
Vistula Kazimierz	–	–	–	–	–	–	–	0.21 (0.06)	–	–	–	0.30 (0.01)	–
Vistula Warsaw	0.40 (0.02)	–	–	–	–	–	–	0.09 (0.01)	–	–	–	<LOQ	–
Vistula Bydgoszcz	–	–	–	–	0.14 (0.03)	–	–	0.19 (0.04)	–	–	–	–	–
Vistula Torun 1	–	–	–	–	0.06 (0.01)	–	–	0.15 (0.04)	–	–	–	0.20 (0.04)	–
Vistula Torun 2	<LOQ	–	–	–	0.12 (0.03)	–	–	0.14 (0.04)	<LOQ	–	–	–	–
Vltava Praga	0.31 (0.06)	–	–	–	0.11 (0.02)	–	–	0.18 (0.03)	–	–	<LOQ	0.50 (0.08)	–
Oder Wroclaw	0.73 (0.15)	–	–	–	0.47 (0.05)	–	0.90 (0.19)	0.14 (0.02)	–	–	–	0.20 (0.01)	–
Warta Zawiercie	–	–	–	–	–	–	–	0.10 (0.03)	–	–	<LOQ	<LOQ	–
Warta Czestoch.	–	–	–	–	0.38 (0.03)	–	–	0.13 (0.02)	0.09 (0.02)	–	–	–	–
Brda Bydgoszcz	–	–	–	–	<LOQ	–	–	0.18 (0.03)	–	–	<LOQ	–	–
Klodnica Gliwice	–	–	–	–	0.07 (0.01)	–	–	0.85 (0.09)	–	–	–	–	–
Krzywa B.–B.	–	–	–	–	0.20 (0.05)	–	–	<LOQ	–	–	–	–	–
Mala Panew Zawadzkie	–	–	–	–	–	–	–	0.11 (0.02)	–	–	–	–	–
Troja Nowa Cerekwia	–	–	–	–	0.17 (0.03)	–	–	0.24 (0.04)	–	–	–	0.19 (0.01)	–
Potok Toszecki Toszek	–	–	–	–	0.32 (0.05)	–	–	–	–	–	–	–	–

A new, rapid and sensitive method has been developed for the simultaneous determination of 13 pharmaceuticals in water samples using an UHPLC-UV. All of the drugs were determined within 3.5 min with satisfactory separations. The low values of LOD and LOQ for most of the drugs allowed for the determination of selected drugs in water samples.

The best recovery efficiencies were obtained with the Oasis HLB column. However, the procedure on each SPE column was applied to the water samples.

Different water samples were analyzed, mostly from Polish rivers from different locations. The drugs used in this study were found in low concentrations (0.06–0.90 μg L^−1^) in all of the water samples.

In conclusion, the SPE-UHPLC-UV method can be successfully applied to the determination of selected drugs in water samples and can be used as a monitoring tool for water pollution in rivers and WWTPs effluents. The method can also be used in laboratories that perform many analyses per day and do not possess expensive LC–MS/MS equipment.

## References

[CR1] Es’haghi Z (2009). Determination of widely used non-steroidal anti-inflammatory drugs in water samples by in situ derivatization, continuous hollow fiber liquid-phase microextraction and gas chromatography-flame ionization detector. Anal Chim Acta.

[CR2] Farre M, Petrovic M, Barcelo D (2007). Recently developed GC/MS and LC/MS methods for determining NSAIDs in water samples. Anal Bioanal Chem.

[CR3] Gil García MD, Cañada Cañada F, Culzoni MJ, Vera-Candioti L, Siano GG, Goicoechea HC, Martínez Galera M (2009). Chemometric tools improving the determination of anti-inflammatory and antiepileptic drugs in river and wastewater by solid-phase microextraction and liquid chromatography diode array detection. J Chromatogr A.

[CR4] Gomez MJ, Martinez Bueno MJ, Lacorte S, Fernandez-Alba AR, Aguera A (2007). Pilot survey monitoring pharmaceuticals and related compounds in a sewage treatment plant located on the Mediterranean coast. Chemosphere.

[CR5] Gracia-Lor E, Sancho JV, Hernandez F (2010). Simultaneous determination of acidic, neutral and basic pharmaceuticals in urban wastewater by ultra high-pressure liquid chromatography-tandem mass spectrometry. J Chromatogr A.

[CR6] Grujic S, Vasiljevic T, Lausevic M (2009). Determination of multiple pharmaceutical classes in surface and ground waters by liquid chromatography–ion trap–tandem mass spectrometry. J Chromatogr A.

[CR7] Hong HN, Kim HN, Park KS, Lee SK, Gu MB (2007). Analysis of the effects diclofenac has on Japanese medaka (*Oryzias latipes*) using real-time PCR. Chemosphere.

[CR8] Kasprzyk-Hordern B, Dinsdale RM, Guwy AJ (2009). Illicit drugs and pharmaceuticals in the environment – forensic applications of environmental data, Part 2: pharmaceuticals as chemical markers of faecal water contamination. Environ Poll.

[CR9] Moldovan Z, Chira R, Alder AC (2009). Environmental exposure of pharmaceuticals and musk fragrances in the Somes River before and after upgrading the municipal wastewater treatment plant Cluj-Napoca, Romania. Environ Sci Pollut Res.

[CR10] Mompelat S, Le Bot B, Thomas O (2009). Occurrence and fate of pharmaceutical products and by-products, from resource to drinking water. Environ Inter.

[CR11] Piram A, Salvador A, Gauvrit JY, Lanteri P, Faure R (2008). Development and optimisation of a single extraction procedure for the LC/MS/MS analysis of two pharmaceutical classes residues in sewage treatment plant. Talanta.

[CR12] Richardson SD (2009). Water analysis: emerging contaminants and current issues. Anal Chem.

[CR13] Rodil R, Quintana JB, Lopez-Mahia P, Muniategui-Lorenzo S, Prada-Rodriguez D (2009). Multi-residue analytical method for the determination of emerging pollutants in water by solid-phase extraction and liquid chromatography–tandem mass spectrometry. J Chromatogr A.

[CR14] Zhou JL, Zhang ZL, Banks E, Grover D, Jiang JQ (2009). Pharmaceutical residues in wastewater treatment works effluents and their impact on receiving river water. J Hazard Mater.

